# The dynamic interplay between *Staphylococcus pseudintermedius* and canine immunity: mechanisms of pathogenesis, evasion, and vaccination perspectives

**DOI:** 10.3389/fcimb.2026.1812880

**Published:** 2026-05-19

**Authors:** Ian Branford, Patrick Butaye, Nwai Oo Khine, Aspinas Chapwanya, Felix N. Toka

**Affiliations:** 1 Center for Integrative Mammalian Research, Department of Biomedical Sciences, Ross University School of Veterinary Medicine, Basseterre, Saint Kitts and Nevis; 2 Department of Infectious Diseases and Public Health, Jockey Club College of Veterinary Medicine and Life Sciences, City University of Hong Kong, Hong Kong, Hong Kong SAR, China; 3 Department of Preclinical Sciences, Institute of Veterinary Medicine, Warsaw University of Life Sciences-SGGW, Warsaw, Poland

**Keywords:** canine immunity, immune evasion, *Staphylococcus pseudintermedius*, vaccine development, virulence factors

## Abstract

*Staphylococcus pseudintermedius* is a common commensal bacterium of dogs. It becomes an opportunistic pathogen when it evades the immune and exogenous antimicrobial control to cause pyoderma, otitis, and postoperative infections in dogs. The shift from colonization to invasive disease illustrates an interplay between bacterial virulence and host immune defense. *S. pseudintermedius* encodes adhesins, exoenzymes, and cytotoxins that facilitate attachment to epithelial surfaces, biofilm formation, and tissue invasion. The bacterium evades the host immune system through IgG-binding proteins, complement inhibition, NET degradation, and modulation of cytokine signaling therefore enabling persistent infection or recurrence, particularly in atopic or immunocompromised hosts. Given that mobile elements found in the genome of *S. pseudintermedius* may encode resistance and virulence genes, these factors may influence selection for lineages that are virulent and methicillin resistant. The animal host defends itself against *S. pseudintermedius* by keratinocyte inflammasome activation, neutrophil recruitment, and Th1/Th17 responses. Although these mechanisms drive bacterial clearance, they can cause tissue injury because of inflammation. Despite increased understanding of *S. pseudintermedius* pathogenesis, effective immunoprophylaxis remains elusive because of bacterial antigenic diversity. Recent research has identified conserved surface proteins and secreted toxins which can serve as candidate antigens for robust vaccine design. This review synthesizes current knowledge on *S. pseudintermedius-*host interactions, highlighting mechanisms of bacterial defense and evasion. Furthermore, we suggest gaps that warrant further research and outline strategies for immunotherapeutics and vaccines against canine staphylococcal infections.

## 1 Introduction

*Staphylococcus pseudintermedius* is a Gram-positive, coagulase- and catalase-positive member of the *Staphylococcus intermedius* group (SIG) and an opportunistic pathogen affecting the skin and mucosae of dogs (Bannoehr and Guardabassi, 2012; Solyman et al., 2013; Priyantha et al., 2016). As recently reported, *Staphylococcus* is the dominant genus among bacterial infections in dogs, accounting for 95.93% of cases. At the species level, *Staphylococcus pseudintermedius* is the most prevalent staphylococcal species, representing 78.89% of isolates (Tanveer et al., 2024). It is partially responsible for canine pyoderma, otitis externa, urinary tract, and postoperative infections. In these infections *S. pseudintermedius* employs surface adhesins, exoenzymes, and cytotoxins to adhere to host cells, evade the immune system, and induce tissue damage (Fitzgerald, 2009). Over the last two decades, methicillin-resistant *S. pseudintermedius* (MRSP) has emerged worldwide as multidrug-resistant (MDR) clones, notably sequence types ST71 and ST68, raising a “One Health” concern due to sporadic zoonotic infections in veterinary personnel and pet owners (Weese and van Duijkeren, 2010; Haller et al., 2025). MRSP was identified in 12 studies, with a pooled prevalence of 64.69% (95% CI: 43.76–81.17) (Tanveer et al., 2024). Diversification of resistance islands and host-adaptive genes continues in MRSP, highlighting its evolutionary flexibility and persistence within canine populations (Grist et al., 2025; Toppi et al., 2025).

Given the growing concern over antimicrobial resistance, gaining a deeper understanding of canine immune responses to *S. pseudintermedius* is essential for developing alternative strategies to control infection. Despite its clinical significance, key host immune pathways, particularly those governing neutrophil recruitment, antibody-mediated neutralization, and Th17-driven mucocutaneous defense, remain incompletely characterized (van Duijkeren et al., 2008; Miller and Cho, 2011). This knowledge gap makes it difficult to develop a vaccine or find immunotherapeutic solutions. However, if we understood the immune mechanisms of protection, it may be easy to create new prophylactic targets, lessen dependence on antibiotics, and mitigate zoonotic risk (Fitzgerald, 2009; Toppi et al., 2025).

However, translating this knowledge into effective interventions is not straightforward. Many conceptual and immunological challenges complicate vaccine development for this commensal opportunist that becomes pathogenic. *S. pseudintermedius* is part of the normal canine microbiota and therefore can be tolerated by the immune system of the dog, which could potentially reduce vaccine efficacy and disrupt microbial balance (Pandey and Khan, 2023; Liu et al., 2024). Another issue is, *S. pseudintermedius* exists as genetically diverse lineages which complicates the selection of universal antigens (Bekeredjian-Ding, 2020). Despite these draw backs, recently, the discovery of antigens such as the immune-evasion proteins (e.g., SpsQ, Luk-I) and conserved adhesion factors (SpsD, SpsL) has created opportunities for multivalent vaccine strategies. The pathogen’s host-specific, yet zoonotic nature, demands balanced protection without collateral inflammation (Abouelkhair et al., 2018b; Elbehiry and Marzouk, 2025). These antigen strategies aim to induce both opsonophagocytic and Th17-mediated memory responses (Abouelkhair et al., 2018b; Abouelkhair et al., 2018a; Balachandran et al., 2018; Lynch and Helbig, 2021; Nocera and De Martino, 2024). Such a dynamic interplay between *S. pseudintermedius* and canine immunity represents a biological challenge on one side and a translational opportunity on the other. Therefore, resolution of this interaction at molecular and immunological levels may lead to development of effective, safe, and long-lasting immunotherapeutics against a microorganism that exists between commensalism and disease.

This review aims to integrate existing knowledge and rigorously examine the dynamic interplay between *S. pseudintermedius* and the canine immune system. We emphasize mechanisms of bacterial pathogenesis, immune evasion, and defense strategies employed by bacteria. Also, we critically examine how bacterial-immune system interactions shape clinical outcomes and contribute to antimicrobial resistance. To provide a framework for the thoughtful development of next-generation vaccines or immunotherapeutics we have discussed the recent advances and ongoing challenges in understanding canine immunity. Ultimately, we strive to bring together fundamental immunology and translational strategies that can help mitigate the growing burden of *S. pseudintermedius* infections in dogs and reduce their zoonotic impact.

## 2 Canine immune response to *Staphylococcus pseudintermedius*

The immune response of dogs to *S. pseudintermedius* is complex and involves both innate and adaptive components that work together to limit bacterial colonization while also having potential to induce inflammation and cause tissue damage.

### 2.1 Innate immune response

Canine β-defensin-103 (cBD103) is a key innate barrier molecule in canine skin, found in the epidermis and adnexal structures where *S. pseudintermedius* colonizes (Leonard et al., 2012). Recombinant cBD103 shows significant bactericidal activity against *S. pseudintermedius* (Fazakerley et al., 2010), paralleling that of human hBD3. However, most data was obtained *in vitro* under non-physiological salt and protease conditions that probably overestimate *in vivo* efficacy. In canine atopic dermatitis (AD) cBD103 expression appears largely preserved compared with healthy skin, in contrast to the reduced defensin levels typical in human AD (Leonard et al., 2012). This supports the concept that functional impairment through mechanisms such as altered secretion, degradation, or peptide inactivation, play a role in *S. pseudintermedius* overgrowth, rather than cBD103 transcriptional loss (Fazakerley et al., 2010). Therefore, it appears that in canine atopic dermatitis, despite similar or increased AMP gene expression, the functional antimicrobial activity of skin surface AMPs against *S. pseudintermedius* is significantly reduced, reflecting impaired host-defense effectiveness even when expression remains intact (Santoro et al., 2011; Santoro, 2018). Staphylococcal resistance mechanisms such as surface charge modification, protease activity, and efflux pumps *(e.g., vraDE)* are well documented in *S. aureus* (Kawada-Matsuo et al., 2021). However, these mechanisms are poorly characterized in *S. pseudintermedius* isolates from dogs, and it highlights a significant gap in the understanding of host-pathogen interaction. Recent work on these innate defensive mechanisms has shifted toward therapeutic exploration. Synergy between cBD103 and chlorhexidine and the efficacy of defensin-derived synthetic peptides against multi-drug-resistant *S. pseudintermedius* (Yang et al., 2018; Santoro et al., 2022) demonstrate translational promise. More recent studies (Ouyang et al., 2024; Stefanetti et al., 2024) extend this by testing novel antimicrobial peptides and reviewing antimicrobial peptides (AMP)-based therapies, whereas Santoro et al. (2022) placed defensins within the wider context of dysbiosis in canine skin. This highlights the absence of current *in vivo* data that directly connects defensin activity to *S. pseudintermedius* colonization. In a murine pyoderma model, peptides such as NZ2114, a plectasin derivative, demonstrated the ability to inhibit *S. pseudintermedius* biofilms and reduced bacterial counts (Zhang et al., 2024). Similarly, synthetic peptides ADD-A and CBD3-ABU (Ouyang et al., 2024) along with short-chain peptides allomyrinasin and andricin B (Tang et al., 2021) showed significant bactericidal, antibiofilm and wound-healing properties while exhibiting minimal cytotoxicity. This underscores the potential of AMP derivatives as promising adjuncts for MRSP management. In our analysis, it is therefore essential for future research to focus on: (1) *in vivo* and *ex vivo* functional assays quantifying cBD103 and related defensins under physiological conditions; (2) proteomic and transcriptomic profiling of *S. pseudintermedius* during its interaction with canine AMPs to define potential species specific resistance mechanisms; (3) comparative investigations of healthy versus atopic skin to distinguish between expression and functional loss of defensins; and (4) controlled therapeutic trials of defensin-mimetic peptides and AMP-antiseptic combinations for managing MRSP. These initiatives are essential for closing the existing divide between the understanding of defensin biology and its practical application in managing canine staphylococcal infections.

Neutrophils represent the principal early responders. They are rapidly recruited to infection sites, where they phagocytose bacteria and utilize reactive oxygen species (ROS) and antimicrobial peptides (Kolaczkowska and Kubes, 2013). Lesions associated with canine pyoderma exhibit significant infiltration of neutrophils and macrophages influenced by cytokines including IL-1β, IL-6, and TNF-α (Hill and Imai, 2016). However, *S, pseudintermedius* possesses several mechanisms to evade or neutralize phagocyte killing. The leukotoxin Luk-I directly lyses neutrophils and macrophages (Abouelkhair et al., 2018a; Balachandran et al., 2018). Additionally, adenosine synthase A (AdsA) converts host DNA-derived AMP to adenosine, which in turn suppresses oxidative burst and phagocytic activity (Abouelkhair et al., 2020). Recent studies demonstrate that virulent *S. pseudintermedius* strains evade neutrophil extracellular traps (NETs) and impair NET-mediated killing *in vivo*, thereby enhancing invasion (Haller et al., 2025). Mast cells and keratinocytes also contribute to early immune signaling. δ-toxin/phenol-soluble modulin (PSM) peptides derived from canine isolates induce mast cell-degranulation and histamine release, establishing a connection between *S. pseudintermedius* colonization to pruritic flares in canine atopic dermatitis (Bell et al., 2025). Keratinocytes respond to *S. pseudintermedius* by activating the NLRP3 inflammasome, leading to IL-1β release and occurrence of pyroptosis (Wang et al., 2024).

The complement system enhances opsonophagocytic clearance. However, *S. pseudintermedius* expresses proteins such as Sbi, which bind immunoglobulins (IgG and IgM) and interact with complement component C3 to inhibit complement activity. *S. pseudintermedius* also expresses SpsQ, a protein A-like IgG-binding surface protein that binds host immunoglobulins. This reduces effective opsonization and potentially hinders complement-mediated bacterial clearance (Sewid et al., 2019; Takeuchi et al., 2023). Structural analysis confirmed that SpsQ binds canine IgG similar to *S. aureus* SpA (Balachandran et al., 2018), revealing a mechanism for host-specific Fc-evasion.

### 2.2 Adaptive immunity

Adaptive immunity in dogs features both T cell-mediated and humoral components. Dendritic cells and macrophages present *S. pseudintermedius* antigens to T cells, promoting Th1 and Th17 responses. The relevant cytokines (IFN-γ, IL-17, IL-22) enhance neutrophil recruitment and bolster epithelial defense (reviewed in (Hill and Imai, 2016)). *S. pseudintermedius* additionally generates superantigens such as SEC-canine, which promotes polyclonal T-cell activation (Carroll et al., 2021). Humoral responses demonstrate significant strength, since dogs affected by atopic dermatitis or staphylococcal pyoderma show increased levels of anti-staphylococcal IgG and IgE (Bexley et al., 2013), with the later suggesting a role for *S. pseudintermedius* antigens in allergic inflammation. Seroproteomic profiling identified several immunodominant proteins, including SpsD, AtpA, FabI and a predicted lipoprotein, which were recognized by IgG in both colonized and infected dogs (Couto et al., 2016). Experimental immunization using recombinant AdsA-M mutants elicits higher IgG titers and increases bacterial phagocytosis by 25-35%, supporting their potential as vaccine candidates (Abouelkhair et al., 2020).

Inspite of these defensive mechanisms, *S. pseudintermedius*’s ability to form biofilms and its strategies for evading the immune response permit it to maintain persistent colonization and lead to recurrent infections. Biofilm-positive isolates demonstrate a stronger virulence-gene expression than commensal strains (Meroni et al., 2019; Andrade et al., 2022; Glajzner et al., 2023).

In summary, the dog immune response to *S. pseudintermedius* is both potent and tightly regulated. It survives innate attacks in form of inflammatory pathways manipulation through adenosine signaling, mast cells activation, and IgG-binding proteins. Future investigations should focus on *in vitro* models that can elucidate these pathways, quantitively map the Th17 and IgE responses, and design of vaccines aimed at conserved immunogenic antigens such as AdsA, SpsQ and Luk-I, to translate these mechanistic insights into effective immunotherapies for canine staphylococcal disease.

## 3 Virulence factors and pathogenesis

*S. pseudintermedius* is a highly adaptable opportunistic pathogen of dogs. It can shift from a commensal colonizer to an invasive pathogen when host defenses are compromised (Bannoehr and Guardabassi, 2012; Hillier et al., 2014; Hill and Imai, 2016; Priyantha et al., 2016; Carroll et al., 2021). Comparative genomic analyses show that virulent lineages often carry expanded accessory genomes. These genomes are enriched with prophages, pathogenicity islands, and resistance determinants, which complement a conserved core of adhesins and toxins (Ferrer et al., 2021; Grist et al., 2025). This genomic flexibility underpins the organism’s ability to adhere to host tissues, evade immune clearance, form biofilms, and withstand antimicrobial pressure. Although these features resemble those of *S. aureus*, they have evolved within a canine-specific immunological context (Bannoehr and Guardabassi, 2012; Bhooshan et al., 2020). **Table 1** provides a concise overview of the virulence factors encoded by *S. pseudintermedius*. While its adhesive properties are increasingly well characterized, quantitative evidence linking specific adhesin expression profiles to disease severity *in vivo* remains limited. This gap highlights an incomplete understanding of *S. pseudintermedius* virulence. The organism also produces a broad array of cytotoxins, suggesting a pathogenic strategy focused on immune disruption rather than stealth. It relies on host-cell lysis and dysregulated cytokine activation to weaken host defenses. Most clinical isolates demonstrate a strong capacity to form biofilms. However, the mechanistic relationships between *ica* operon activity, toxin regulation, and the persistence of chronic infections have not been fully validated *in vivo* and require further investigation.

**Table 1:** *S. pseudintermedius* virulence factors.

Virulence factor category	Specific factors	Pathogenic mechanism
Adherence and Tissue Binding	Cell Wall-Associated Proteins (CWA): SpsD, SpsL, SpsO, SpsQ (Protein A)	Adhesion & colonisation (MSCRAMMs, LPXTG-anchored proteins, *sps*/*sdr* genes): Surface-anchored proteins such as fibronectin-binding proteins (*fnbA, fnbB*), clumping factors (*clfA, clfB*), the *sps* family (e.g., *spsE, spsO, spsP, spsQ, spsL, spsD*), *sdrC/E*, *lipA*, and the sortase-A enzyme (*srtA*) mediate binding to extracellular matrix proteins (fibronectin, fibrinogen, cytokeratins) and are present in the great majority of isolates (Manco et al., 2006; Bannoehr et al., 2011; Bannoehr and Guardabassi, 2012; Janesch et al., 2018; González-Martín et al., 2020; Zukancic et al., 2020; Carroll et al., 2021; Hisirová et al., 2025; Horsman et al., 2025; Usui et al., 2025). SpsD and SpsL are known to facilitate invasion of canine epithelial cells (Bannoehr and Guardabassi, 2012; Pietrocola et al., 2015). Genes for SpsD, SpsF, SpsP, and SpsQ have been found predominantly in pyoderma isolates (Ferrer et al., 2021).
Pore-Forming Toxins (PFTs)	Luk-I Leukotoxin (*lukS/F* genes) (Bannoehr et al., 2007; Abouelkhair et al., 2018a).	Specifically targets and lyses canine polymorphonuclear cells (PMNs), crippling the innate immune response (Abouelkhair et al., 2018a; Bhooshan et al., 2020; Carroll et al., 2021). Toxin repertoire - Exfoliative toxin (*speta*) - induces epidermal desquamation, Enterotoxins (*se-int, siet*) - superantigen activity, provoke T-cell proliferation, Leukocidins (*lukF, lukS, LukD, LukE*) - pore-forming toxins that lyse neutrophils and macrophages, and Hemolysins (β-haemolysin *hlb*, α-haemolysin absent in some strains) and *sec* (enterotoxin C) - damage host cells and promote inflammation (Becker et al., 2001; Futagawa-Saito et al., 2009; Iyori et al., 2011; Maali et al., 2018)..
	Phenol-Soluble Modulins (PSMs): δ-toxin, PSM-ε (Maali et al., 2018)	Induce direct, receptor-independent membrane disruption and lysis of non-professional phagocytic cells (e.g., osteoblasts, keratinocytes) (Maali et al., 2018).
Secreted Toxins	SpEX exotoxin	Inhibits neutrophil chemotaxis, impairs complement-mediated clearance, cytotoxic to immune cells (Abouelkhair et al., 2019).
Secreted Proteases	Pst metalloprotease (aur-like)	Thermolysin-family protease; tissue degradation, nutrient acquisition, immune modulation (Wladyka et al., 2008).
Enzymes/Biofilm	Coagulase (prCoa) (Sewid et al., 2019)	Activates host prothrombin to deposit a protective fibrin shield around the bacteria, inhibiting phagocytosis (Sewid et al., 2018). *lip2* (lipase) and *hlg* genes (γ-haemolysin) contribute to tissue degradation; *staphyloferrin* siderophores aid iron acquisition (Ben Zakour et al., 2011);.
	*icaA*, PNAG, *agr* quorum sensing, *icaADBC*	Formation of an extracellular polymeric substance (EPS), often involving upregulation of the *icaA* gene (Crawford et al., 2014), which creates a physical barrier that resists antibiotics and host defenses (Tang et al., 2021; Rana et al., 2024; Zhang et al., 2024). The *icaADBC* operon synthesizes poly-N-acetyl-glucosamine (PNAG), the main matrix of staphylococcal biofilms. Biofilm producers are classified as strong, moderate or weak; most clinical isolates are strong producers (≥ 80 %). The *agr* system regulates dispersal and toxin expression (Alonzo et al., 2012; Cabral et al., 2025; Chan et al., 2025; Hisirová et al., 2025; Owaku et al., 2025; Saengsawang et al., 2025; Savaliya et al., 2025) (Garbacz et al., 2013; Tang et al., 2021; Nocera and De Martino, 2024; Zhang et al., 2024).
Exfoliative Toxins (ETs)	EXI (ExpA) (González-Martín et al., 2020).	Selectively digests canine desmoglein 1 (Dsg-1), causing characteristic epidermal splitting and subcorneal clefts in canine skin (Nishifuji et al., 2008; Iyori et al., 2011).
	Enterotoxin (SECcanine) (Banchereau et al., 2012; Carroll et al., 2021).	Distinct from human enterotoxins, capable of inducing vomiting and T-cell proliferation (Youn et al., 2011; Tanabe et al., 2013; Carroll et al., 2021; Lee et al., 2023).
Superantigens	SEC_canine	Polyclonal T-cell activation and cytokine steorm; associated with dermatitis flares (Yoon et al., 2010; Lee et al., 2023).
Immune evasion (Anti-NETs/Anti-phagocytic)	NucB + AdsA (SpsAdsA)	NucB degrades NETs; AsdA converts dAMP to deoxyadenosine killing phagocytes (Bünsow et al., 2021).
Immunoglobulin and complement evasion	Sbi paralogs	Bind IgG Fc/Fab, IgM and C3, blocking opsonization and complement activation (Sewid et al., 2019).
Structural Virulence Factors	Wall Teichoic Acid (WTA) biosynthesis genes	Host adaptation, modulation of immune recognition, complement interference (??) (Winstel et al., 2014).

The persistence of *S. pseudintermedius* is further enhanced by immune evasion strategies that neutralize innate host defenses. These mechanisms enable the bacterium to convert host defense by-products into cytotoxic metabolites, promoting intracellular survival and facilitating dissemination. Capsular polysaccharides also contribute by inhibiting complement activation and reducing opsonophagocytosis (Morais et al., 2025; Saengsawang et al., 2025). However, much of the available evidence is derived from genomic analyses or *in vitro* studies, and the extent to which these factors are expressed during natural infection remains unclear.

The most prevalent MRSP clones worldwide, ST71 and ST45, are major contributors to canine disease. These clones harbor prophage-associated virulence genes, including *virE*, *BacSp222*, *mecA*, *blaZ*, *qac*, and *cadA.* The convergence of antimicrobial resistance and enhanced virulence suggests that therapeutic pressure may favor the emergence of hypervirulent phenotypes (Goncheva et al., 2019; Freitas et al., 2025; Grist et al., 2025; Seo et al., 2025; Su et al., 2025). These MRSP lineages also exhibit increased biofilm-forming capacity and enhanced immune evasion. The resulting complexity in treating chronic infections, such as pyoderma, underscores the need for coordinated antimicrobial stewardship. This should be coupled with genomic surveillance to mitigate the risk of further increases in virulence.

At the cellular level, *S. pseudintermedius* modulates host signaling pathways to sustain infection. In canine corneal epithelial cells, infection activates the ROS-NLRP3 inflammasome-caspase-1 cascade, leading to gasdermin D-mediated pyroptosis and subsequent tissue necrosis (Wang et al., 2024). Although this inflammatory response is intended to control infection, it can paradoxically exacerbate pathology and facilitate bacterial dissemination by promoting tissue damage. These findings highlight a delicate balance between host defense and self-injury, in which *S. pseudintermedius* exploits the host’s own inflammatory processes to enhance its pathogenic potential.

The pathogenesis of *S. pseudintermedius* arises from a complex interplay of adhesive, cytotoxic, enzymatic, and immunomodulatory mechanisms embedded within a genetically adaptable genome. Its ability to simultaneously colonize, resist immune clearance, and exploit host defenses distinguishes it from coagulase-negative staphylococci. Despite substantial genomic insight, functional studies in canine models remain limited. Key priorities include defining the temporal expression of virulence factors *in vivo*, understanding their regulation within biofilms, and characterizing their interactions with the canine immune system. Addressing these gaps represents a critical research frontier. Such integrative approaches will be essential for translating virulence profiling into effective vaccines or therapeutics. Targeting conserved proteins such as SpsD, AdsA, Luk-I, and SpsQ, which play central roles in adhesion, immune evasion, and persistence, offers promising avenues for intervention. Insights gained from this work may also inform the development of *S. aureus* vaccines, which have yet to achieve consistent clinical success. In this context, the canine model of *S. pseudintermedius* may provide a valuable platform for advancing human vaccine research.

## 4 Immune evasion mechanisms

*S. pseudintermedius* persists in canine tissues by coordinating three overlapping layers of immune evasion: (i) disruption of primary innate defenses, (ii) bypassing the antibody-complement axis, and (iii) fortifying itself behind physical and phenotypic shields that blunt host effector responses and limit therapeutic efficacy. The interplay of these mechanisms likely contributes to the persistence and recurrence of clinical conditions such as pyoderma, otitis, and post-surgical infections. Moreover, antimicrobial use may further select for resistant strains, promoting the emergence of MRSP as a significant One Health concern. Recent genomic surveillance studies and case series have also documented zoonotic spillover alongside increasing antimicrobial resistance, thereby amplifying the clinical impact on both canine and human health (Guardabassi et al., 2013; Gaffen et al., 2014; Abouelkhair et al., 2018a; Suepaul et al., 2023; Myrenås et al., 2024; Viñes et al., 2024).

### 4.1 Disruption of innate defenses

Frontline sabotage centers on the interplay between nuclease activity and adenosine production, and how these processes drive leukocytotoxicity. During abscess formation, *S. pseudintermedius* secretes the major nuclease NucB, which degrades host DNA, including NETs. This degradation releases deoxyribonucleotides that AdsA subsequently converts into cytotoxic deoxyadenosine (dAdo). The resulting dAdo selectively kills macrophages and promotes bacterial persistence. Both NucB and AdsA are widely distributed across global lineages, with strong support from *in vivo* and *in vitro* studies (Bünsow et al., 2021). In parallel*, S. pseudintermedius* produces a bicomponent leukotoxin (LukS-I/LukF-I, collectively “Luk-I”) that lyses canine neutrophils, further weakening innate immune defenses at infection sites (Maali et al., 2018). At the same time, a cell-wall-anchored AdsA ortholog (SpAdsA) dephosphorylates extracellular ATP into adenosine. This adenosine then signals through A2A receptors on host phagocytes, suppressing their bactericidal activity. Inhibition of SpAdsA or blockade of A2A receptors restores killing capacity in canine whole blood and neutrophil assays, highlighting a tractable therapeutic target (Abouelkhair et al., 2020). Together, these findings underscore the central role of NucB/AdsA-mediated innate immune suppression. They also point to actionable intervention strategies, such as nuclease or AdsA inhibition, and adenosinergic antagonism, that warrant translational evaluation in dogs.

### 4.2 Antibody-complement interference

*S. pseudintermedius* disrupts the antibody-complement axis through Ig-binding proteins and direct interference with complement activity. The protein A homolog SpsQ binds the Fc region of IgG, effectively inverting opsonins on the bacterial surface. By reorienting antibodies, SpsQ prevents Fc receptor recognition and blocks opsonization and phagocytosis. In addition, SpsQ can engage VH3-family B-cell receptors in a superantigen-like manner. This interaction leads to deletion or functional inactivation of B-cell clones, ultimately reducing antigen-specific responses, like the effects described for *S. aureus* SpA (Abouelkhair et al., 2018b). Complement pathways are targeted by Sbi paralogs. *S. pseudintermedius* encodes Sbi-like proteins that bind canine IgG/IgM as well as complement component C3. These interactions impair convertase function through factor H-C3-mediated mechanisms, thereby demonstrating direct complement antagonism and disrupting innate-adaptive immune crosstalk (Sewid et al., 2019). Despite the strength of these protein-level mechanisms, *in vivo* studies examining complement activation kinetics, such as C3b deposition and C5a gradient formation in canine tissue infections remain limited. This gap constrains biomarker development and hampers the rational application of complement-modulating therapies.

### 4.3 Biofilm and physical shielding

Physical and phenotypic shielding represent the third layer of *S. pseudintermedius* immune evasion. Biofilm formation is a key component of this strategy and is partly driven by the *icaADBC* locus, which encodes the poly-N-acetyl-β-(1→6)-glucosamine/polysaccharide intercellular adhesin (PNAG/PIA) matrix. This extracellular matrix limits antimicrobial penetration and restricts access by immune effectors, including antibodies, complement proteins, and phagocytes. As a result, it promotes bacterial persistence, particularly in device-associated and mucocutaneous infections. Recent studies reaffirm the central role of biofilms in pathogenesis and emphasize their regulation by global systems such as agr and SarA. These regulatory pathways may represent exploitable therapeutic targets, as highlighted in recent reviews (Nocera and De Martino, 2024; Elbehiry and Marzouk, 2025).

Coagulase produced by *S. pseudintermedius* activates prothrombin, leading to the formation of a fibrin shell around the bacteria. This structure shields the pathogen from leukocyte engagement and represents a classic immune-evasion mechanism shared among staphylococci. Evidence of this activity in *S. pseudintermedius* suggests that antithrombin or anti-coagulase approaches could serve as useful adjunctive therapies (Sewid et al., 2018). In parallel, the accumulation of resistance determinants, such as *mecA*, *blaZ*, and antiseptic tolerance loci, indirectly enhances immune evasion by enabling prolonged carriage and persistence under therapeutic pressure. Microbiome disruption may further contribute, as susceptible commensal populations are eliminated during treatment. Genomic surveys support these concerns. They document the expansion of clonal MRSP, widespread multidrug resistance, and evidence of transmission between pets and their owners (Frosini et al., 2019; Myrenås et al., 2024; Horsman et al., 2025).

### 4.4 Critical analysis and synthesis

What implications does this have for intervention? First, several *S. pseudintermedius*-specific targets have demonstrated proof-of-concept: (i) nuclease/AdsA inhibition to block dAdo-mediated phagocyte killing; (ii) antagonism of the adenosine pathway (A2A) to restore neutrophil/monocyte function; (iii) neutralization of SpsQ/Sbi to re-enable opsonophagocytosis and complement activation; and (iv) anti-biofilm and anti-coagulase agents to dismantle physical barriers. The nuclease-adenosine axis presents a compelling case due to the alignment of *in vitro* data and genetic conservation (Bünsow et al., 2021). Second, surveillance studies should explicitly track immune evasion genes *(adsA/nucB, spsQ, sbi, lukS/F-I, icaADBC, coa)* alongside resistance loci to link genotypes with outcome in canine cohorts and to flag lineages that combine hard-to-treat phenotypes with potent immune escape (Futagawa-Saito et al., 2004; Sewid et al., 2018; Meroni et al., 2019; Sewid et al., 2019; Bünsow et al., 2021; Srednik et al., 2023; Takeuchi et al., 2023; Myrenås et al., 2024). Third, the most notable gaps of knowledge remain e.g., the need for real-time mapping of complement activation/evasion *in vivo*, strain-resolved roles of Luk-I and PSMs during canine infections, and the necessity for controlled trials evaluating adenosinergic and anti-coagulase strategies, as supplementary measures to decolonization or antibiotics (Futagawa-Saito et al., 2004; Maali et al., 2018; Pitchenin et al., 2018; Sewid et al., 2018; Sewid et al., 2019; Bünsow et al., 2021; Cheung and Otto, 2023; Cheung et al., 2024).

The mechanism of toxin-mediated neutrophil killing (Luk-I) and nucleotide-toxin chemistry (NucB/AdsA) indicate that, *S. pseudintermedius* targets the first hours of infection, particularly regarding phagocyte recruitment and NET formation. This suggest that early innate immune neutralization is the decisive battleground. This convergence explains why mobile elements carrying these functions are so strongly selected in invasive clones (Abouelkhair et al., 2018b; Abouelkhair et al., 2018a; Bünsow et al., 2021). In sum, the success of *S. pseudintermedius* in dogs illustrates a program that degrades NETs and macrophages (NucB/AdsA), neutralizes opsonins and complement (SpsQ/Sbi), and conceals itself within biofilms and fibrin structures while AMR extends the timeframe for all these mechanisms. This description effectively captures the pillars of successful infection and newer research supports adenosinergic immunosuppression and complement interference. Additionally, extensive genomic analysis highlights the critical need to integrate antimicrobial stewardship with immunologically informed countermeasures (Sewid et al., 2019; Abouelkhair et al., 2020; Myrenås et al., 2024). Translation to *S. aureus* is possible as they share many characteristics. There is a need for further research, especially *in vivo*, the dog model offers opportunities for this critical information as *S. aureus* is and remains a major human pathogen for which vaccines have yet to be developed.

## 5 Bacterial defense and genomic adaptation

The evolutionary success of *S. pseudintermedius* as both a commensal and opportunistic pathogen is attributed to its sophisticated defensive structure. It has preserved genomic integrity while supporting persistence within the host. *S. pseudintermedius* encodes a complex system of “innate immunity” against foreign DNA, primarily through restriction-modification (RM) systems (Type I/II, sometimes III) and lineage-variable CRISPR-cas, along with phage insertions that may inhibit natural competence (e.g., within comG). Together, these systems regulate horizontal gene transfer (HGT), controlling the entry and persistence of various plasmids, transposons, and prophages. In successful epidemic lineages of *S. pseudintermedius*, such as ST71, a prophage (SpST71A) disrupts comGA, thereby effectively switching off DNA uptake. These same lineages also show RM/CRISPR patterns that are associated with reduced recombination and a “closed” accessory genome. Functionally, that means immune evasion arsenals become more established and fixed overtime, whereas barrier-light lineages remain open to acquiring new mobile immune modulators. This mechanistic framework is strongly supported by population-genomic studies and experimental genetics (Brooks et al., 2020; Phumthanakorn et al., 2021). **Table 2** presents defense and adaptation systems for major lineages of *S. pseudintermedius* along with their corresponding sequence types (ST).

**Table 2:** **A** summary of defence/adaptation-systems for major lineages of *S. pseudintermedius* with ST, sequence type, known defence system features (R-M, CRISPR/Cas, competence disruption).

ST (agr)	Geography	R-M systems	Defense genes on SCCmec(examples)	Other defense features (chromosomal/prophage	Key evidence
ST71 (agr III)	Europe-dominant MDR clone	SCCmec II-III / III	Type-I RM module within SCCmec in multiple ST71 genomes	Prophage SpST71A disrupts comGA (competence); lineage shows reduced recombination; CRISPR uncommon	(McCarthy et al., 2015; Brooks et al., 2020; Srednik et al., 2023; Morais et al., 2025)
ST68	North America MDR clone	SCCmec V(T) (and variants)	Reports of CRISPR and Type-I/II RM cassettes carried on SCCmec in ST68	Diverse prophages; barrier profile differs from ST71	(McCarthy et al., 2015; Phumthanakorn et al., 2021)
ST45	Europe/Global	ΨSCCmec57395	RM modules reported with SCCmec; CRISPR generally rare	No specific comG disruption emphasized	(Chanchaithong et al., 2016; Srednik et al., 2023; Morais et al., 2025)
ST261	Europe (emergent)	SCCmec IV	RM present on SCCmec IV in comparisons vs ST71	–	(McCarthy et al., 2015)
ST258	N. Europe emergence replacing ST71 in some settings	Mixed (often SCCmec IV/V; lab/region-dependent)	RM modules frequently; CRISPR uncommon	Clonal replacement noted in population work	(Damborg et al., 2016; Bergot et al., 2018)
ST496	Often “commensal-leaning” vs disease	Variable; often SCCmec IV/V or MSSP	Fewer RM (I/IV) than disease-linked clones; CRISPR Type IIA/IIIA reported (chromosomal)	–	(Sawhney et al., 2023) (Grist et al., 2025)
ST551	Less common; includes MRSP/MSSP	SCCmec V(T)SL/154 reported	CRISPR Type IIC / IIIA with SCCmec V(T)SL/154 in exemplar strain; multiple RM	–	(Morais et al., 2025)
ST64	Reported in global sets	SCCmec IV/V (region-dependent)	Evidence of Type-I + Type-II RM combo on SCCmec (one methylase incomplete)	–	(Bruce et al., 2022)
ST84	Sporadic	SCCmec IV/V	Type-I/II RM on SCCmec	–	(Bruce et al., 2022)
ST1183	Recent report	SCCmec V(T)SL/154; plus fusC cassette elsewhere	RM common; CRISPR rare	–	(Morais et al., 2025)
ST261-like cluster (AU)	Australia cluster	Novel SCCmec variants described	Some composite SCCmec with RM/metal-resistance modules (e.g., co-carried SCCczrAI16-CI)	–	(Chanchaithong et al., 2016; Worthing et al., 2018)
Mixed (multi-ST panels)	Diagnostic vs commensal vs household	III most frequent, plus IVg, V(T), Ψ57395, novel cassettes (e.g., SCCmec7017-61515)	RM prevalent (mostly Type-I) across strains; CRISPR in ~18–30%, sometimes adjacent to SCCmec	Niche-specific selection on defense genes; many intact prophages	(Sawhney et al., 2023; da Silva et al., 2024; MacFadyen and Paterson, 2024; Morais et al., 2025)

Key sources. McCarthy 2015 (McCarthy et al., 2015): side-by-side maps of SCCmec II-III (ST71) vs SCCmec IV (ST261) vs V(T) (ST68) and presence of RM modules on SCCmec; Brooks 2020 (Brooks et al., 2020): SpST71A prophage - comGA disruption in ST71, lower recombination in clones with CRISPR or competence disruption; lineage-specific prophages; Chanchaithong 2016 (Chanchaithong et al., 2016): Composite SCCmec with co-located RM/metal-resistance (SCCczrA) modules in MRSP from Thailand (illustrates defense cargo in SCC elements); Srednik 2023 (Srednik et al., 2023): concrete ST71-SCCmec III and ST45-ΨSCCmec57395 case reports; Sawhney 2023 (Sawhney et al., 2023): niche-specific selection of defense genes; Type-IIIa CRISPR in mecA+ isolates, sometimes adjacent to SCCmec; da Silva 2024 (da Silva et al., 2024): panel with CRISPR present in ~18% (mostly MSSP), SCCmec III most frequent; confirms variable carriage of defense systems.; Morais 2025 (Morais et al., 2025): 17-genome mobilome analysis; lists SCCmec types (III, IVg, V(T)SL/154, Ψ57395, NA45, SCCmec7017-61515), widespread RM Type-I, CRISPR in 5/17 (often MSSP), and exemplars like BIOS-V227 with IIC/IIIA CRISPR plus SCCmec V(T)SL/154; MacFadyen 2024 (MacFadyen and Paterson, 2024): compiles emerging novel SCCmec including SCCmec7017–61515 in S. pseudintermedius; Phumthanakorn 2021 (Phumthanakorn et al., 2021): same-ST dog/human MRSP genomes; diverse prophages and pathogenicity islands - context for defense vs MGE flux.

RM, restriction–modification; T I/II/III/IV, RM type; “CRISPR on SCCmec”, cas genes encoded adjacent to/within SCCmec; “ΨSCCmec”, pseudo-SCCmec lacking ccr.

### 5.1 Genetic “border control” mechanisms

The numerous potent host-modulating factors encoded on prophages or associated with other mobile elements indicate that defense configurations play a crucial role in determining the range of immune evasion strategies available to a clone. A prime example is the bicomponent leukotoxin LukS-I/LukF-I, encoded on degenerate prophage lineages that allow for prophage carriage or exhibit reduced barriers are more likely to gather these leukocyte-killing modules, and this has clear implications for their survival against phagocytic clearance (Abouelkhair et al., 2018a). *S. pseudintermedius* also utilizes an extracellular “nuclease-adenosine” axis, NucB and adenosine synthase A, to transform host DNA into deoxyadenosine, thereby selectively impairing macrophages during abscess formation. This strategy, initially characterized in *S. aureus*, is now well known in MRSP and appears to be broadly distributed across the species exposing the interaction between fundamental virulence mechanisms and the evasion of innate immunity (Bünsow et al., 2021). The genetic accessibility of these loci, and their regulation on mobile contexts connects their dissemination to the state of lineage-specific barriers.

Large scale genomic analysis reveals geographically structured accessory gene pools, featuring intact RM systems in many strains and detectable CRISPR-cas in a subset. The presence of RM systems and CRISPR-cas correlated with methicillin resistance of many MDR isolates (Brooks et al., 2020; Bruce et al., 2022; Cheung et al., 2024; Myrenås et al., 2024; Morais et al., 2025; Zehr et al., 2025). These data suggest repeated, environment-specific adjustment in defense genes, which is consistent with the idea that clinical contexts favor the selection for defense configurations. The presence of intact RM systems and occasional CRISPR-cas implies multiple evolutionary strategies in order to maintain a genome stability against adaptability (Morais et al., 2025).

Two additional advances help clarify how genome plasticity links to immune evasion. First, neutralization of Luk-I remains a promising avenue for canine-targeted vaccine development, given that recombinant Luk-I proteins have been shown to elicit neutralizing responses and the corresponding genes are highly prevalent across *S. pseudintermedius* isolates (Abouelkhair et al., 2018a). More broadly, if prophage-encoded toxins are major contributors to disease severity, then two complementary strategies could, in principle, shift population-level virulence: (a) immunization targeting these toxins to blunt their pathogenic effects, and (b) ecological or evolutionary interventions that limit acquisition or maintenance of toxin-encoding prophages by increasing the fitness cost of permissive defense states. While the former is a tractable vaccinology objective, the latter remains a longer-term research goal that depends on better understanding of how bacterial defense systems such as restriction-modification and CRISPR-Cas influence horizontal gene transfer and prophage dynamics in staphylococci (Marraffini and Sontheimer, 2008). Secondly, high resolution surveillance studies reveal that what traditionally has been referred to as a “dog pathogen” is genomically diverse across hosts and environments, with diagnostics now detecting broader host range and niche-associated accessory genes. The observed heterogeneity probably indicates distinct defense system configurations shaped by different ecological pressures, which in turn influence the immune-evasion modules that are retained (Sawhney et al., 2023).

### 5.2 Critical analysis and synthesis

*S. pseudintermedius* balances between gene acquisition and stability by utilizing RM/CRISPR and competence disruption as key mechanisms of evasion. Clones that “close the borders” establish a robust, albeit less adaptable, immune evasion toolkit. But those that stay open and can sample new phage and/or plasmid cargo and occasionally acquire leukotoxicins or enzyme systems, can counteract innate canine defenses. However, these come with the trade-off of genomic burden and potential fitness liabilities. Recent genomic analysis shows that *S. pseudintermedius* maintains multiple equilibria rather than one optimal solution (Brooks et al., 2020; Zehr et al., 2025). Phage therapy or plasmid-based delivery must navigate RM/CRISPR landscapes, advocating for customized vectors matched to lineage-specific barriers. Population genomics provide the atlas needed to do this rationally (Morais et al., 2025; Zehr et al., 2025).

In summary, *S. pseudintermedius*’s genetic defense and adaptation mechanisms are more than a mere “anti-phage locks”, they serve as primary regulators of the species’ immune evasion capabilities. In environments with strict defenses, immune evasion becomes deeply rooted and consistent; conversely in more relaxed settings, strategies frequently shift towards increasingly aggressive tactics aimed at undermining neutrophils and macrophages. This ecological and evolutionary perspective is consistent with the genomic and functional studies (Abouelkhair et al., 2018a; Brooks et al., 2020; Bünsow et al., 2021).

## 6 Vaccine development strategies and antigen targets

The increasing occurrence of MDR and MRSP continues to erode therapeutic options in canine staphylococcal infections, making vaccination a priority for both prophylaxis and adjunctive therapy (Abouelkhair et al., 2018b; Abouelkhair et al., 2018a; Balachandran et al., 2018; Abouelkhair et al., 2020). Recent epidemiological findings highlight the prevalence (Tanveer et al., 2024) and persistent presence of SSC*mec* mediated methicillin-resistance across various clonal lineages (e.g., ST71, ST68) and its increasing zoonotic potential. This emphasizes the necessity for strategies aimed at mitigating disease severity and minimizing antibiotic use, rather than striving for sterilizing immunity against this commensal, present in close to over 70% of the dogs (Nocera and De Martino, 2024; Roberts et al., 2024; Tanveer et al., 2024).

A consensus is emerging that the most promising targets are conserved factors related to virulence and immune evasion. The non-toxigenic protein A homolog mutant (SpsQ-M) retained antigenicity yet lost B-cell apoptogenic and superantigenic activities. It successively elicited neutralizing antibodies in dogs, which reduced immunosuppressive effects of wild type SpsQ *in vitro*, thus positioning it as a rational component of a vaccine or immunotherapeutic (Abouelkhair et al., 2018b; Balachandran et al., 2018). These findings currently hold strongly and serve as fundamental basis.

Leukotoxin Luk-I represents a significant antigen of interest. Dogs immunized with attenuated subunits (Luk-I-M, Luk-F-M) generated neutralizing antibodies that reduced toxin-mediated killing of canine neutrophils. This outcome serves as an essential functional indicator associated with preserved innate defense (Abouelkhair et al., 2018a). Similarly, the cell wall 5’-nucleotidase AdsA (SpAdsA) is a compelling immune evasion target. Antibodies elicited by an attenuated SpAdsA enhanced opsonophagocytic clearance by canine whole blood, consistent with the enzyme’s role in adenosine-mediated immunosuppression (Abouelkhair et al., 2020). The identified classes of antigens specifically antitoxin and anti-evasion correlate directly with mechanisms of significance at the lesion, thereby strengthening the biological plausibility of protection (Abouelkhair et al., 2018a; Abouelkhair et al., 2020).

It is important to note that relying on a protein A-only strategy carries inherent risks. There is significant plasticity at the SpsQ (*spa*) locus. About 62% of global isolates completely lack *spa*, and this locus serves as recombination “hot spot” that incorporates unrelated cargo, undercutting any vaccine that heavily might rely on the presence or uniformity of SpsQ (Zukancic et al., 2020). Therefore, SpsQ although not yet validated experimentally, could be maintained as part of larger combination rather than used as a standalone antigen. Focusing on adhesins is appealing as they tackle the initial stages of disease. SpsD and SpsL are microbial surface components recognizing adhesive matrix molecules (MSCRAMMs) that facilitate adherence to canine corneocytes and fibrinogen/keratin, a function shown in various experimental systems, and they elicit humoral responses in naturally infected dogs (Bannoehr et al., 2011). A vaccine component that can prevent SpsD/SpsL-mediated adhesion may hinder colonization, decrease inoculum at would sites, and synergize with antitoxin immunity (Bannoehr et al., 2011; Bannoehr and Guardabassi, 2012; Pietrocola et al., 2015).

### 6.1 Metabolic antigens

Proteomics screens consistently reveal conserved metabolic proteins (e.g., AtpA, FabI) that are essential for *in vivo* survival of *S. pseudintermedius*. Several studies have emphasized the importance of incorporating these proteins into multivalent constructs, which could ensure effectiveness across the various clonal backgrounds (Couto et al., 2016; Jaan et al., 2022; Stefanetti et al., 2024). Although, antibodies induced by these antigens might not directly neutralize toxins, they could contribute to a diverse immune repertoire and could enhance opsonophagocytic killing. Recent reviews suggest combining “offense” (antitoxin/anti-evasion) with “sustainment” (metabolic/core fitness) antigens to mitigate the effects of antigenic flux (Couto et al., 2016; Hemmadi and Biswas, 2021; Shinwari et al., 2025). These approaches look promising but await experimental validation.

The ongoing setbacks of *S. aureus* vaccines in humans have shifted the focus to understanding the nature of protective immunity. Effective defense against staphylococcal skin infections in preclinical models requires both functional antibodies and Th1/Th17 responses, which are essential for activating and directing neutrophils to the site of infection (Miller and Cho, 2011; Montgomery et al., 2014). For *S. pseudintermedius*, this argues for adjuvant systems that favour IL-1/IL-17-linked cutaneous immunity while minimizing the risk of immunopathology. This is a flexible yet attainable objective with modern adjuvants and delivery platforms as suggested for *S. aureus* (Miller and Cho, 2011; Montgomery et al., 2014; Ferraro et al., 2019; Mills, 2023).

### 6.2 Biofilm/PNAG considerations

Considering the biofilm ecology of recurrent pyoderma, dPNAG-like polysaccharide antigens are still intriguing as potential cross-staphylococcal components that could facilitate opsonophagocytic killing and biofilm disruption. While most PNAG research is in *S. aureus* and related species, recent advancements in synthetic glycan libraries have improved epitope selection and demonstrated strong preclinical protection in mice. These technologies have the potential to be adapted for canine MRSP with appropriate feasibility studies (Brooks and Jefferson, 2014; Mirzaei et al., 2021; Tan et al., 2024; Liao et al., 2025). The scientific argument is reasonable, however, there is still a lack of direct data regarding the *S. pseudintermedius* PNAG vaccine, and the phase variation of PNAG may affect consistency. PNAG ought to be considered as a modular component rather than a fundamental antigen.

Because *S. pseudintermedius* is a commensal, achieving complete sterilization of mucosal immunity may not be practical and could even be counterproductive as it is an inherent part of the healthy microbiota, therefore, the immediate objectives are (i) to lessen lesion severity, (ii) to decrease disease duration, and (iii) to minimize antibiotic use. The retrospective studies of autogenous staphylococcus bacterins in dogs suggest a decrease in antimicrobial prescriptions in recurrent pyoderma, although the quality of these studies varies and the underlying mechanisms remain ambiguous (Wilson et al., 2019). These data support clinical trial endpoints centered on antibiotic-sparing benefits and relapse reduction rather than colonization.

In our opinion, an effective canine vaccine concept includes the following components: (1) core antigens that are mechanism-led-attenuated Luk-I-M/LukF-I-M) combined with attenuated SpAdsA-M to enhance neutrophils protection and restore phagocytic function (Abouelkhair et al., 2018a; Abouelkhair et al., 2020); (2) adhesin anchor SpsD/SpsL domains aimed at minimizing early tissue adhesion and load (Bannoehr et al., 2007), and (3) optional adjuncts, non-toxigenic SpsQ-M (for strains that express it) and a PNAG-derived epitope module to introduce opsonophagocytic killing against biofilm-embedded bacteria, recognizing variability and pending *S. pseudintermedius*-specific validation (Bannoehr and Guardabassi, 2012; Wilson et al., 2019; Zukancic et al., 2020; Tan et al., 2024). We envisage formulation and immunology that utilizes an adjuvant/delivery system that skews toward a Th1/Th17 response, while maintaining the production of high quality opsonophagocytic antibodies, e.g., TLR-agonist-containing emulsions or liposomes with depot effect for skin. Preclinical immune readouts must include opsonophagocytic killing against the various sequence types, toxin neutralization assays on canine neutrophils, and skin site cytokine signatures (IL-17A/F, IFN-γ) in dogs (Miller and Cho, 2011; Montgomery et al., 2014). Trial designs could prospectively enroll dogs with recurrent superficial pyoderma/otitis, using antibiotic sparing and time-to-relapse as primary endpoints, in addition to safety and microbiome monitoring to evaluate commensal perturbation (Wilson et al., 2019; Loeffler et al., 2025).

There is still no licensed canine vaccine against *S. pseudintermedius*, and variability at the spa/SpsQ locus confirms the caution against single-antigen strategies. However, the functional advancement tailored for dogs such as neutralizing Luk-I, and disarming AdsA to limit Sps-mediated adhesion, present a cohesive, multivalent framework that aligns with our understanding of *S. aureus* regarding the importance of synchronized antibody and Th1/Th17 responses. The next step in this area involves conducting integrated canine trials that prioritize functional correlates such as toxin neutralizing and opsonophagocytic killing, along with pragmatic clinical outcomes like reduced antibiotics use and fewer relapses, rather than solely focusing on colonization clearance. This approach aligns with the standards expected of a vaccine targeting a common commensal turned opportunist.

## 7 Discussion and future perspectives

*Staphylococcus pseudintermedius* occupies a unique niche serving as a canine commensal while also demonstrating significant adaptability as an opportunistic pathogen. The clinical success of *S. pseudintermedius* stems from a multifaceted strategy integrating surface adhesion, cytotoxicity, metabolic immunomodulation, complement interference, and biofilm-mediated persistence. These mechanisms are reinforced by a genetically flexible mobilome shaped by lineage-specific restriction modification systems, CRISPR-Cas elements, and prophage integration. Simultaneously, canine immunity mounts robust innate and adaptive responses, characterized by defensin activity, complement involvement, neutrophil recruitment, and Th1/Th17 responses. However, these defenses are intentionally compromised by *S. pseudintermedius* factors like Luk-I, SpAdsA, SpsQ and Sbi paralogs that directly neutralize or disrupt essential immune pathways. This interplay between bacterial and host processes establishes a dynamic and reciprocal relationship that influences colonization, onset of disease, chronicity, and relapse.

Despite significant progress, several limitations continue to create important knowledge gaps in this field. Current understanding of *S. pseudintermedius* pathogenesis remains fragmented, particularly at the interface between bacterial virulence and host immunity. A central gap is the incomplete characterization of key host immune pathways, including those governing neutrophil recruitment and neutrophil-*S. pseudintermedius* interactions, antibody-mediated neutralization, and Th17-driven mucocutaneous defense. Although these processes are fundamental to infection control, their coordination, kinetics, and functional outcomes in the canine host remain insufficiently defined. These uncertainties have direct clinical implications. Inadequate understanding of neutrophil and antibody function limits the development of effective immunotherapies and vaccines, while gaps in Th17-mediated immunity constrain strategies aimed at improving mucocutaneous barrier protection, particularly in dogs with recurrent or atopic infections. In addition, the *in vivo* dynamics of complement activation, such as C3b deposition and C5a gradient formation, remain poorly understood, hindering the identification of reliable biomarkers of disease activity and the rational design of complement-targeted interventions. Addressing these gaps will be essential for advancing mechanism-based diagnostics, improving patient stratification, and enabling the development of targeted immunomodulatory and anti-virulence therapies.

Limitations also extend to innate antimicrobial defenses. There are no robust *in vivo* and *ex vivo* assays quantifying cBD103 and other AMPs, nor sufficient insight into their functional activity in diseased versus healthy skin. Comparative studies are needed to distinguish between reduced expression and functional impairment of defensins during atopic infections. In parallel, integrated proteomic and transcriptomic profiling of *S. pseudintermedius* during interaction with canine AMPs is required to define species-specific resistance mechanisms. These gaps have direct clinical implications. The absence of functional AMP assays limits the development of biomarkers for barrier immunity and hinders the identification of dogs at risk of recurrent infection. Similarly, without distinguishing defensin deficiency from dysfunction, targeted therapeutic strategies, such as AMP replacement or immunomodulation, cannot be rationally applied. A better understanding of bacterial resistance to AMPs would also inform the design of anti-virulence therapies and help predict treatment failure, particularly in MRSP infections. Further, the lack of controlled therapeutic studies evaluating defensin-mimetic peptides or AMP-antiseptic combinations restricts clinical translation. Addressing these gaps could enable the development of novel topical or adjunctive therapies aimed at restoring innate barrier function, reducing antibiotic reliance, and improving outcomes in chronic and drug-resistant infections.

At the pathogen level, genomic studies have mapped the distribution of virulence and immune evasion determinants across *S. pseudintermedius* lineages. However, the temporal expression, regulation, and *in vivo* activity of these factors during natural infection remain poorly defined. Key priorities include elucidating how virulence programs are dynamically regulated, particularly within biofilms, and how they interact with host immune responses over the course of infection. These gaps have important clinical consequences. Without understanding when and where virulence factors are expressed, it is difficult to identify optimal therapeutic windows or to design targeted anti-virulence interventions. Similarly, incomplete knowledge of biofilm-associated regulation limits the development of effective anti-biofilm strategies and contributes to treatment failure in chronic and device-associated infections (also relatively under-characterized in dogs). More broadly, the field lacks integrative studies linking bacterial genotype to host immune phenotype and clinical outcomes in dogs. This disconnection constrains the development of predictive biomarkers, hampers patient stratification, and limits the implementation of precision medicine approaches. Addressing these gaps will be essential for translating genomic insights into clinically actionable diagnostics and targeted therapies.

## 8 Conclusion

*S. pseudintermedius* is a highly adapted opportunistic bacteria that can evade the immune system, form biofilms, and resist antimicrobials in dogs. Future control of *S. pseudintermedius* require approaches beyond antibiotics alone. The bacterium also causes recurrent pyoderma, otitis, wound infections, and postoperative complications. Given zoonotic transmission and shared resistance determinants, it has become a One Health zoonotic concern. Pathogenesis is driven by leukotoxins and nuclease-adenosine pathways impairing innate immunity, and immunoglobulin-binding and complement-modulating proteins weakening opsonization. Biofilm formation and coagulase-mediated shielding promote persistence and chronic infection. Thus, control strategies ought to include vaccines, anti-virulence therapies, biofilm disruption, host-directed immunomodulation, and improved antimicrobial stewardship. Methicillin-resistant *S. pseudintermedius* reduces treatment options. Despite genomic advances, key translational gaps remain since neutrophil responses, Th17-mediated mucocutaneous defense, complement kinetics, and antimicrobial peptide function, are still not fully understood. The temporal regulation of virulence factors during natural infection warrants further research, particularly within biofilms. Interactive studies are warranted to connect bacterial genotypes, host immune phenotype and clinical outcomes. Deeper understanding of this may require systems immunology, functional microbiology, comparative genomics, and clinical phenotyping systems approach in dogs.
